# Outpatient Management of the Hematopoietic Stem Cell Transplant Patient

**DOI:** 10.6004/jadpro.2016.7.3.16

**Published:** 2016-04-01

**Authors:** Sandra Kurtin, Ali McBride

**Affiliations:** University of Arizona Cancer Center, Tucson, Arizona

Hematopoietic stem cell transplant (HSCT) is increasingly used for patients with hematologic malignancies, with about 20,000 performed each year in the United States. Advanced practitioners care for these patients across the disease trajectory, and should be aware of the early and late effects of HSCT and the prophylactic measures to keep them healthy, according to clinicians from the University of Arizona Cancer Center, Tucson, who described the outpatient management of HSCT patients at JADPRO Live at APSHO.

"After day 100, the transplant patient may be back in your practice," said Sandra Kurtin, RN, MSN, AOCN®, ANP-C. The advanced practitioner will be responsible for many aspects of care, including diagnosing infection, identifying relapse, screening for and preventing late effects, managing graft-vs.-host disease (GVHD), coordinating psychosocial support, and referring to other care collaborators, she said.

Joining Ms. Kurtin in the presentation was Ali McBride, PharmD, MS, BCPS, BCOP, who discussed the immunosuppressed state and prophylaxis against infections.

According to Ms. Kurtin, "Early referral to a transplant center is perhaps the single most important factor that will affect survival," therefore, she urged clinicians to consider the possibility of transplant early in the work-up of patients with hematologic malignancies.

"Timing for referrals is crucial. If transplant may be an option, ask questions early, get started on eligibility and insurance issues," she advised.

## DETERMINING TRANSPLANT ELIGIBILITY

Survival varies according to disease state, tempo of the disease, general fitness of the patient, and age.

Clinicians determine eligibility for transplant by evaluating the disease state, previous treatments, previous infections, transfusion history, comorbidities and performance status, which is essentially a "fit vs. frail" question, Ms. Kurtin said.

The comorbidity index is informative, since patients with three uncontrolled comorbidities have been shown to have inferior outcomes. "This may be a deal breaker in how you move forward," she said. "It’s important for clinicians to manage these comorbidities, to maintain eligibility."

A psychosocial evaluation is also essential, as "this is a life-changing event," she noted. "It’s intensive and hard, even in the best of situations."

HSCT is also very expensive, which is a reason to start the conversation early with third-party payers. If there is push-back, this process can take months, she indicated.

## STEM CELL DONOR CRITERIA

Autologous transplants are used for myeloma and lymphomas. All myeloid malignancies require an allogeneic transplant.

Donors for an allo-HSCT can be either related donors or unrelated donors. Unrelated human leukocyte antigen (HLA)-matched donors are found through the National Marrow Donor Registry. While a full match is optimal, "that’s driven by what’s available in the registry." And, "finding matches is particularly challenging for ethnic minorities," she added.

## HSCT FOR AML

Ms. Kurtin described the HSCT process for patients with acute myelogenous leukemia (AML). Transplant is considered important for patients at risk for relapse, including patients who achieve a first complete remission (CR) but have unfavorable features, with antecedent hematologic disease (e.g., myelodysplastic syndrome), patients with treatment-related leukemia, patients who relapse soon after induction, patients with minimal residual disease after treatment, and patients in second CR and beyond if not previously evaluated for HSCT. 

Survival differs based on the timing of transplant. In a recent study, about 50% of patients undergoing HSCT in first or second CR were alive at 5 years, compared to about 20% with advanced disease (Vyas, Applebaum, & Craddock, 2015).

## COMMON COMPLICATIONS OF HSCT

Post-HSCT symptoms can vary according to intensity of the conditioning regimen and degree of immunosuppression, but generally the most common early effects are fatigue, disturbed sleep, drowsiness, lack of appetite (which can lead to weight loss and "withering"), and physical weakness. Symptoms typically peak at around day 30, according to a study from MD Anderson Cancer Center (Cohen et al., 2012). Guidelines for monitoring early and late complications for HSCT can be found at BeTheMatch.org.

Acute and chronic GVHD are both common after allogeneic transplant. Chronic GVHD begins to emerge when patients are tapered off immunosuppressive agents. Most organ systems can be affected (Table 1). Early detection of chronic GVHD is important for preventing irreversible organ damage and improving survival.

**Table 1:**
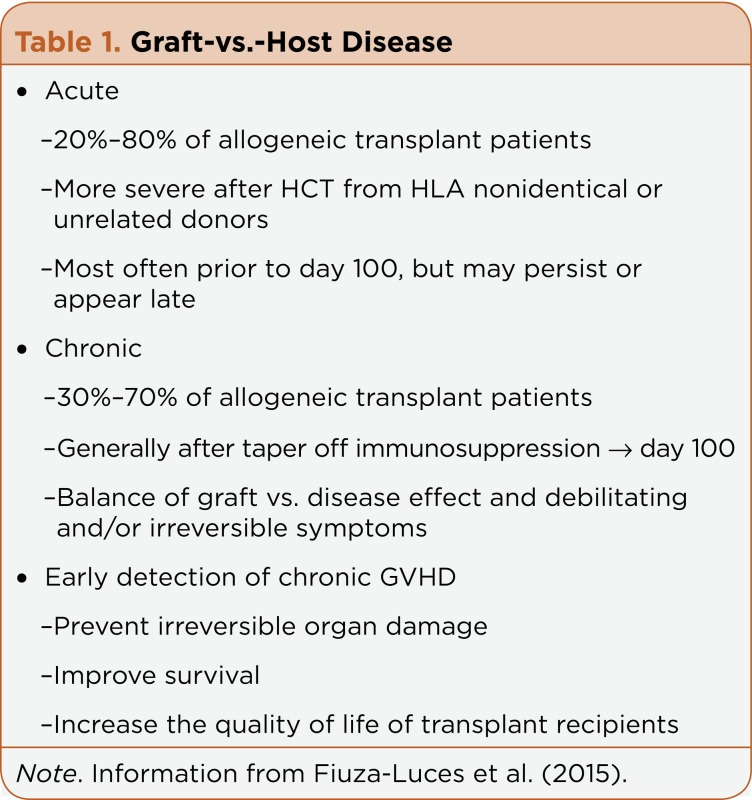
Graft-vs.-Host Disease

"Stay connected to the transplant center for ongoing management of these conditions," Ms. Kurtin advised.

## CONSEQUENCES OF IMMUNOSUPPRESSED STATE

Dr. McBride discussed B-cell and T-cell immunity and the immunosuppressed state created by the HSCT process. There are several phases of immune suppression corresponding to the preengraftment phase, post-engraftment and late phase of transplant, and they are associated with different organisms and different opportunistic infections. For days 0 through 30 post-HSCT, there is recovery of neutrophils and phagocytic function. At 6 months post-HSCT, B cells gain function and immune globulin levels normalize. At 12 months, cellular immunity develops and immune constitution returns.

To varying degrees throughout the post-HSCT course, HSCT patients are susceptible to neutropenia, lymphopenia, hypogammaglobulinemia, viral infections, and bacterial infections. Patients with chronic GVHD have an increased risk of invasive infection, vs. those without GVHD, because of maximum immunosuppression that allows opportunistic infections to emerge.

He outlined what clinicians can expect and should do for each phase.

- Neutropenic phase (2-4 weeks post-HSCT): Patients lack an effective immune system and are very susceptible to infection. Supportive care and empirical antibiotics are "mainstays of a successful passage through this phase," he said.
- Engraftment phase: Healing begins. Mucositis resolves, fevers subside, infections begin to clear. Management of GVHD and prevention of viral infections, especially cytomegalovirus (CMV), are important. Monitoring for CMV during and after immunosuppression should be done weekly, and prophylaxis can be considered.
- Post-engraftment phase: This phase lasts months to years. The hallmarks are gradual development of tolerance and weaning off of immunosuppression, management of chronic GVHD, and laboratory documentation of immune reconstitution.

For autologous transplant patients, he recommended HSV antiviral prophylaxis with acyclovir 800 mg bid; antifungal and prophylaxis for encapsulated bacteria are typically not required.

Allogeneic transplants may require a broader spectrum of prophylactic coverage, including fungal, pneumocystis jiroveci pneumonia, and herpes simplex prophylaxis. In addition, CMV titers via polymerase chain reaction are often used for monitoring of CMV reactivation, vs. that of CMV prophylaxis. Further monitoring with EBV in haplo transplants may also be considered.

## VACCINATIONS

"There is loss of antibody titers to vaccine-preventable diseases during the first 4 years after HSCT," he pointed out. These infections include mostly Streptococcus pneumoniae, Haemophilus influenza type b, influenza A, and varicella zoster.

Dr. McBride suggested that clinicians regularly refer to a vaccine titer schedule that spans 6 to 60 months posttransplant, calling this a "key piece" of the management protocol (Table 2).

**Table 2:**
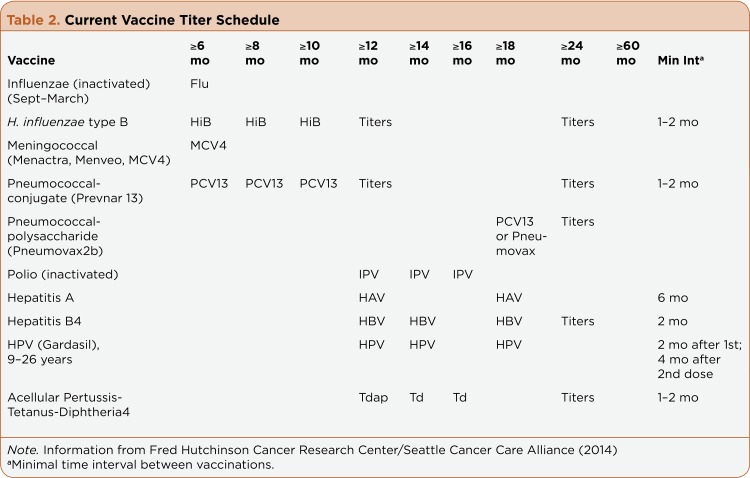
Current Vaccine Titer Schedule

In addition, he offered several practical recommendations for vaccinations:

- Tetanus: Successful immunization against tetanus is achieved with 3 doses of vaccine, regardless of GVHD status. If given at 6 months, patients achieve 90% of immunity.
- Diphtheria: Loss of antibodies can be seen after HSCT; multiple doses of vaccine are the best means of prevention.
- Haemophilus influenza type b (HIB): This infection occurs approximately 90 days post-HSCT. Early immunization (3 to 6 months) produces high levels of titers, vs. later vaccination (12 months), but the efficacy appears to be similar. If the donor has been vaccinated, the HSCT recipient has enhanced protection.

Finally, Dr. McBride emphasized that care of the transplant patient may become even more prolonged, as maintenance regimens are gaining favor, at least in multiple myeloma and lymphoma, and these carry their own toxicity profiles.
